# Supplementary material for: Persistent extrasynaptic hyperdopaminergia in the mouse hippocampus induces plasticity and recognition memory deficits reversed by the atypical antipsychotic sulpiride
Source: PLoS One. 2023 Aug 25;18(8):e0289770. doi: 10.1371/journal.pone.0289770 (PMC10456148; doi:10.1371/journal.pone.0289770)
Supplement: Attachment — Submitted filename: Response to the reviewers.pdf [file pone.0289770.s003.pdf]

Reviewer #1:

This is a beautiful, novel and elegant study that I loved reading, providing several unexpected observations that are tempting food for thought. This is probably the reason for several of the queries listed below:

We are grateful to this reviewer, Dr Rodrigo A. Cunha who is a recognized specialist of purinergic transmission and neuropsychiatric disorders, for his very positive comments.

1- Spatial reference memory has been mostly associated with information processing in the dorsal rather in the ventral aspects of the hippocampus. Instead, the ventral part of the hippocampus is mostly involved in the emotional state and emotional-related memories. Thus, there seems to be a need to discuss the apparently paradoxical effects on spatial reference memory upon selective manipulations of the ventral hippocampus. Moreover, it seems relevant to enquire if fear memory, namely extinction of fear memory, and anxiety-/depressive-like behaviors are not modified with these dopamine-related manipulations in the ventral hippocampus. With the data already collected, some information may be obtained, namely by quantifying the relative time in the center and periphery of the open field and some thigmotaxic behavior in the Morris water maze. Indeed, this clarification of eventual GBR-induced emotional alterations seems important since alterations of mood-like behavior could explain differences in the spatial-memory tests.

We have now added a paragraph and several references to the Discussion. In addition, we have now included the relative time in the center and periphery of the open field and the thigmotaxic time in the Morris water maze in the body of the manuscript and as part of the supplementary figure 3.

A growing number of studies in recent years have suggested the existence of a functional continuum, related to spatial processing and navigation, along the dorsoventral hippocampal axis. Loureiro and colleagues demonstrated that participation of the ventral hippocampus is substantial for the expression of a spatial memory representation. In a pioneering study, Fredes et al., 2021 validated such functional continuum from the dorsal to the ventral hippocampus, demonstrating that mossy interneurons in the ventral dentate gyrus detect environmental novelty and that this information is conveyed to dorsal dentate granule cells and is necessary for novelty-induced contextual memory formation. Interestingly, we previously showed that mossy cells of the dentate gyrus constitute the main D2 receptors expressing cells in the ventral hippocampus. Considering a positive correlation between the activity of ventral mossy interneurons on one hand and environmental novelty and contextual memory acquisition on the other hand, it is conceivable that suppression of ventral mossy cells due to over-activation of their D2 receptors by dopamine excess would lead to impairment in spatial memory formation in ventrally infused mice. In support of this hypothesis, antagonism of D2 receptors by the antipsychotic sulpiride successfully rescued the spatial memory deficit induced by chronic GBR infusion.

2-The title explicitly claims that the study explores a ‘Persistent Extrasynaptic Hyperdopaminergia’. Please provide the evidence for this blunt conclusion that this reviewer could not reach after reading the manuscript.

In the introduction, we are now providing more detailed evidences for the rapid and long lasting effects of DAT blockade by the GBR compounds on extracellular dopamine.

While we did not perform microdialysis in our study, GBR 12935 has long been established as the most selective DAT inhibitor amongst the GBR family with a high affinity for DAT (Kd~10nM), although very closely related to GBR12909 and GBR12921 in structure. In vivo microdialysis in the striatum or the nucleus accumbens of GBR12935 as well as systemic or local administration of GBR12909 show a rapid (within 20 minutes) and long-lasting increase of extracellular DA.

3-The mentioning of effects of extrasynaptic dopamine prompts questioning if DAT is present in astrocytes and/or microglia in the hippocampus and if dopaminergic effects on these glial cells can be excluded as a possible mechanism of GBR-induced electrophysiological and behavior effects.

In the Discussion section we are now providing some background and references on a possible, but probably very limited, role for DAT activity within glial cells.

DAT has been suggested to be expressed by astrocytes and microglia *in vitro*, although it is not established if glia in the hippocampus also express DAT. In our hands, DAT staining was limited to neuronal fibers in the ventral Hippocampus and any glial staining fell below our detection threshold. Therefore, while we believe our observations mainly originates from the neuronal component, we cannot exclude a possibility of glial DAT contribution, to a certain extent, to the effects of GBR on electrophysiology and behavior.

4-The title also claims that the modifications caused by GBR treatment were 'Reversed by Antipsychotics'. This is a rather group of drugs and only a D2 receptor-preferring antagonist was tested out of the several drugs in this class. This may need to be reviewed.

We thank the reviewers for their pertinent comment and agree with him that our study could not imply "all" typical and atypical antipsychotics. Title was corrected to reflect the fact that sulpiride was only tested in the present manuscript, as follow:

Persistent Extrasynaptic Hyperdopaminergia in the Mouse Hippocampus Induces Plasticity and Recognition Memory Deficits Reversed by the Atypical Antipsychotic Sulpiride.

5-p.4 'GBR12935 has a high specificity for DAT blockade'. Specificity means that a drug only has one target irrespective of the concentration. Thus, specificity is not high or low, instead it exits or does not exist. Unfortunately, there is no single drug that is specific, they are all at best selective. Correct.

We thank the reviewers for this pertinent comment. Specificity accordingly was changed to selectivity in the manuscript.

6-p.5: At least define the hippocampal synapses where the recordings were done as well as the recording medium in the core of the manuscript.

Recordings were performed on CA3-CA1 glutamatergic synapses using artificial CSF for electrophysiological recordings. This was added under Materials and Methods section.

7-p.5 ‘GraphPad (Prism, which version?)’: I am afraid only the authors will be able to answer this question.

GraphPad Prism 6.02 and 8 were used in the study. The version have now been mentioned in Statistical Comparisons.

8-p.10 ‘Time spent exploring the novel item (object or place)’: I may have missed it, but I could not find the description of the control values during the training period of the treated and untreated mice: did they spend a similar total time exploring the two objects during the training phase?

We have now included the relative times the mice from both groups spent exploring the left and right object (NORT) and place (OPRT) in the body of the manuscript on page 10, under “Chronic GBR12935 infusion abolished recognition memory and induced pruning of DA fibers” and as part of the supplementary figure 3.

Mice from both groups displayed no preference towards either left or right object or place on the training day (Figure Sup. 3E, F). The observed spatial memory deficits were not simply a consequence of emotional alterations following chronic GBR infusion into the ventral CA1, as the total distance and relative times spent in the center and periphery were not significantly different between GBR- and NaCl-treated mice when a subset of animals were tested in an open field (Figure Sup. 3A-C). In keeping with this, relative thigmotaxis time was similar between GBR-infused and NaCl control mice, in spite of a significant lower rate of successful trials by GBR-treated mice on day 9 of the spatial MWM (Figure Sup. 3D).

9-p.12 ‘Figure 5. In vitro acute effect of GBR12935 (30nmol/L) on LTP and LTD in ventral CA1’: I would strongly advise the authors to include representative fEPSP recordings to convince the readers (and this reviewer) of the quality of the recordings, especially in the presence of bicuculline, which tends to result in heavily contaminated fEPSPs, even without knowing the constitution of the recording solution, which I would consider the minimal detail that should be provided in the core manuscript.

We were unfortunately unable to retrieve the original fEPSP recordings, and we are sorry for that. Original traces were kept in a computer that have been since reformatted and these data have been unfortunately deleted. We could have provided fEPSP from other set of experiments but did not wanted to do so. Though, it should be said that all recordings have been done under the control and watch of Dr Tak-Pan Wong in our institute (co-author on this Ms), who is an outstanding and dedicated electrophysiologist with decades of practice in that field.

10-p.13 ‘memory, GBR-treated animals displayed a hyper-exploratory phenotype, possibly due to a deficit in long-term habituation’: I could not find the data sustaining this statement. It seems mandatory to request seeing these data since alterations of locomotion can be sufficient to explain differences in the memory-related behavior tests.

We have included exploratory behavior of both groups in the open field as part of the supplementary figure 3. As shown in panel A, GBR-treated mice displayed a very mild hyper locomotion, which did not reach significance level (p=0.668). Therefore, we removed this sentence from Discussion.

11-p.13 ‘memory, GBR-treated animals displayed a hyper-exploratory phenotype, possibly due to a deficit in long-term habituation’: This also raises the question of defining the searching strategy of mice without or with treatment with GBR (see Garthe and Kempermann, 2013, Front. Neurosci. 7, 63).

Please see the response to comment # 10.

12-p.14 ‘Hippocampal lesions generally spare short-term recognition memory performance (50)’: This contention is at least debatable since several acute interference with hippocampal function result in profound impact on short term memory tests (60-90 min in OD or OR).

We agree with the reviewer and removed this sentence from Discussion.

Reviewer #2:

Rocchetti et al. described in this manuscript their study on the effect of persistent extrasynaptic hyperdopaminergia in the mouse hippocampus, which induces plasticity and recognition memory deficits on behavior using behavior tests and electrophysiology. They concluded that antagonizing D2Rs was beneficial for cognitive functions in the context of hippocampal hyperdopaminergia.

Although the study appears complete by its data, several major concerns include deficient experimental design and misused statistical methods, missing critical information on technical details, invalidated behavioral data, and failure to put their findings in the context of relevant non-therapeutic-oriented studies in the literature.

Major concerns:

It is necessary to provide evidence for how well the repeated administration model of GBR 12935, a dopamine transporter (DAT) blocker in the hippocampus, simulates psychiatric disorders in clinical settings???

Somehow, this reviewer is much more far-reaching than we are in the conclusion of our study, limited to an animal model of local hyperdopaminergia. That is probably our fault, and we write now in the introduction:

In the present study, we did not aim or claim to mimic clinical conditions. Given the subcortical hyperdopaminergia theory of schizophrenia (1, 2), our goal was to investigate how localized hyperdopaminergia in the mouse hippocampus would affect memory-associated behavior in mice. In addition, in light of our previous findings on the detrimental role of D2 blockade on mesohippocampal synaptic transmission and on hippocampus-associated learning and memory under normodopaminergic condition (22), we studied if an antipsychotic, with D2 antagonism as its putative mechanism of action, could restore some of the cognitive impairments observed with hippocampal hyperdopaminergia.

Behavioral changes can occur when dopamine function is enhanced, and results indicating indirect cognitive impairment may be obtained depending on the behavioral tests. These results naturally depend on the extracellular dopamine levels and are thought to be antagonized by antipsychotic drugs. For example, in some patients with schizophrenia, brain dopamine function is enhanced, but compensatory dopamine receptor function is thought to be decreased if dopamine levels are always high. In this animal model, it should be shown what functional changes occur due to repeated administration of GBR. For example, how long after discontinuing the infusion of the DAT inhibitor for 2-3 weeks did extracellular dopamine levels increase, and how much did dopamine neuron reactivity change before the next infusion? Additionally, it should be confirmed that there were no histological impairments.

Regarding the long-lasting effects on functional change, the stronger argument is presented on Figure 3F, where we have a significant decrease of DAT fibers after chronic GBR without any histological impairments. In addition, and thanks to the reviewer for noticing this omission, we now report in the Supplemental section, under “Intrahippocampal infusions”:

24 hours after the last day of behavior experiment, mice were euthanized, and their brain removed for histological examinations. Mice with visible hippocampal lesions (about 5%) were removed from any analysis.

The effects of GBR compounds are now discussed in the introduction.

While we did not perform microdialysis in our study, GBR 12935 has long been established as the most selective DAT inhibitor amongst the GBR family with a high affinity for DAT (Kd~10nM), although very closely related to GBR12909 and GBR12921 in structure. In vivo microdialysis in the striatum or the nucleus accumbens of GBR12935 as well as systemic or local administration of GBR12909 show a rapid (within 20 minutes) and long-lasting increase of extracellular DA.

Since it has yet to be known whether behavioral experiment data are close to the normal distribution, there may be problems presenting results with mean values and standard errors, Student t-tests that assume a normal distribution. At the very least, each data point should be shown as a scattered dot blot on the graph.

We thank the reviewer for this very legitimate request. All datasets in our study passed D'Agostino & Pearson normality test (we have added this under Statistical comparisons); therefore, they follow a normal distribution. For more clarity, we changed all the column bar graphs in figures 2-4 to scatter plots with bar.
